# Evaluating Seasonal Variations in Human Contact Patterns and Their Impact on the Transmission of Respiratory Infectious Diseases

**DOI:** 10.1111/irv.13301

**Published:** 2024-05-11

**Authors:** Allisandra G. Kummer, Juanjuan Zhang, Chenyan Jiang, Maria Litvinova, Paulo C. Ventura, Marc A. Garcia, Alessandro Vespignani, Huanyu Wu, Hongjie Yu, Marco Ajelli

**Affiliations:** ^1^ Laboratory for Computational Epidemiology and Public Health, Department of Epidemiology and Biostatistics Indiana University School of Public Health Bloomington Indiana USA; ^2^ Shanghai Institute of Infectious Disease and Biosecurity, Department of Epidemiology, School of Public Health Fudan University Shanghai China; ^3^ Department of Epidemiology, School of Public Health Fudan University, Key Laboratory of Public Health Safety, Ministry of Education Shanghai China; ^4^ Shanghai Municipal Center for Disease Control and Prevention Shanghai China; ^5^ Lerner Center for Public Health Promotion, Aging Studies Institute, Department of Sociology, and Maxwell School of Citizenship & Public Affairs Syracuse University Syracuse New York USA; ^6^ Laboratory for the Modeling of Biological and Socio‐technical Systems Northeastern University Boston Massachusetts USA

**Keywords:** human contact patterns, mathematical modeling, respiratory pathogens, seasonality

## Abstract

**Background:**

Human contact patterns are a key determinant driving the spread of respiratory infectious diseases. However, the relationship between contact patterns and seasonality as well as their possible association with the seasonality of respiratory diseases is yet to be clarified.

**Methods:**

We investigated the association between temperature and human contact patterns using data collected through a cross‐sectional diary‐based contact survey in Shanghai, China, between December 24, 2017, and May 30, 2018. We then developed a compartmental model of influenza transmission informed by the derived seasonal trends in the number of contacts and validated it against A(H1N1)pdm09 influenza data collected in Shanghai during the same period.

**Results:**

We identified a significant inverse relationship between the number of contacts and the seasonal temperature trend defined as a spline interpolation of temperature data (*p* = 0.003). We estimated an average of 16.4 (95% PrI: 15.1–17.5) contacts per day in December 2017 that increased to an average of 17.6 contacts (95% PrI: 16.5–19.3) in January 2018 and then declined to an average of 10.3 (95% PrI: 9.4–10.8) in May 2018. Estimates of influenza incidence obtained by the compartmental model comply with the observed epidemiological data. The reproduction number was estimated to increase from 1.24 (95% CI: 1.21–1.27) in December to a peak of 1.34 (95% CI: 1.31–1.37) in January. The estimated median infection attack rate at the end of the season was 27.4% (95% CI: 23.7–30.5%).

**Conclusions:**

Our findings support a relationship between temperature and contact patterns, which can contribute to deepen the understanding of the relationship between social interactions and the epidemiology of respiratory infectious diseases.

## 1 Introduction

Several respiratory infectious diseases, including influenza and respiratory syncytial virus (RSV), show clear seasonal trends and cyclic epidemics [1, 2, 3]. Temperate regions experience higher incidence during the winter season with fewer cases occurring during the summer months, whereas places with tropical climates, such as Singapore, may observe a higher incidence in warmer months or year‐round [1, 3, 4, 5, 6, 7]. Possible explanations for the seasonality of respiratory infectious diseases include the variation in meteorological conditions (e.g., absolute humidity, temperature) that influence virus transmission, virus survival, and host susceptibility [5, 8, 9]. Seasonal changes have also been linked to changes in host immune function; particularly decreases in mucosal integrity during dry seasons may increase susceptibility to infection [4, 6]. Previous studies have also indicated that influenza virus survival is associated with water vapor in the air, suggesting that absolute humidity (a measurement of water vapor regardless of temperature) might be a driver of the seasonality of influenza epidemics seasonality [8, 9]. This finding is supported by some epidemiological studies [8, 9, 10], but not by others [11, 12, 13], suggesting that this may be a contributing factor but is not the sole driver of influenza seasonality.

Human contact patterns are crucial for understanding the epidemiology of respiratory infectious diseases, as they are a key determinant in infectious disease transmission [5, 14, 15, 16, 17]. Highest rates of transmission have been reported in social settings where individuals are in close contact with others, such as households, schools, and workplaces [5, 18, 19, 20]. Contacts made in these settings are typically consistent throughout the year; however, human behavior adapts to contextual changes such as working days, weekend days, holidays, weather conditions, and season [5]. Reductions in the number of contacts among school‐age children during school closures were shown to be associated with reductions in influenza cases during holiday breaks [13, 14, 15, 18]. Furthermore, people tend to spend more time indoors when the temperature drops [5, 21], increasing an individual's proximity to others, thus impacting their likelihood of contracting an infectious disease [22, 23]. Broadly, temporal variations in human behavior may be a contributing factor in seasonal changes in disease incidence [24].

In this work, we investigate whether human contact patterns follow seasonal trends and the extent to which these trends contribute to shaping influenza seasonal patterns. To this aim, we collected contact survey data, meteorological data, influenza‐like illness (ILI) data, and A(H1N1)pdm09 influenza positivity rates for Shanghai, China, during the 2017–2018 influenza season. We conducted a regression analysis showing significant associations between contact patterns and temperature. We then leveraged the obtained results to calibrate a mathematical model of influenza transmission, which is validated against the collected ILI data for Shanghai. Ultimately, the performed simulations showed temporal changes in the reproduction number as temperature fluctuates seasonally. These findings suggest that, in the study location, contact patterns follow a seasonal trend, which may be a contributing factor to the observed seasonality of influenza epidemics.

## 2 Methods

### 2.1 Data

#### 2.1.1 Contact Survey

The current study is based on data collected from 965 individuals in Shanghai, China, who participated in a cross‐sectional diary‐based contact survey conducted from December 24, 2017, to May 30, 2018. A full description of the contact survey can be found in Zhang et al. [25] and contact diaries are available on Zenodo [26]. To recruit participants into the study, 40 Shanghai neighborhoods were randomly selected. In each neighborhood, convenience sampling was used to select 25 households. Then, one household member was randomly selected to participate in the study until the number of participants for each age group and for each gender planned during the survey design was reached. A day of the week was sampled from a uniform distribution and assigned to each participant to complete a self‐reported questionnaire. The questionnaire collected individual demographic and socioeconomic information of the study participants along with the number of persons with whom they had contact during the 24 h prior to completing the contact survey, the date when contacts occurred, and details of each contact (i.e., relationship, location, duration, and type). Contacts were defined as either a two‐way conversation exchanging three or more words in the physical presence of another person or a skin‐to‐skin contact. The original contact patterns study was approved by the ethical review committee at the School of Public Health at Fudan University, Shanghai, China (Ref: 2018‐01‐0659S). Informed consent was obtained from all subjects (from a parent or guardian if the participant was under 18 years of age).

#### 2.1.2 Meteorological Data

Meteorological data from December 1, 2017, to May 30, 2018, for Shanghai were obtained from the Hongqiao International Airport Station using an online historical archive of weather reports [27]. Maximum daily temperatures were matched to the calendar dates when participants completed their contact diaries for the statistical analysis. Maximum temperature was chosen for the primary analysis because minimum temperature occurs during the night when people are least active, and their contacts are less likely to be affected by those temperatures. Average daily temperatures, which reflect the dynamics of both the maximum and the minimum temperatures and the daily absolute humidity, were used in sensitivity analyses to assess the robustness of the findings of the main analysis.

#### 2.1.3 Influenza Surveillance

We obtained weekly reports of total ILIs and laboratory‐confirmed influenza infections, as well as the number of total specimens tested for influenza viruses, from Shanghai CDC for the 2017–2018 influenza season. These weekly reports include both inpatient and outpatient ILI cases of all ages. In the 2017–2018 season, there were 30 sentinel hospitals in Shanghai, including 19 national and 11 municipal sentinel hospitals. In each national sentinel hospital, nasopharyngeal swabs were collected from ILI patients and placed in sterile viral transport medium for influenza virus testing. ILI was defined as temperature ≥ 38°C with either cough or sore throat, in the absence of an alternative diagnosis. As a result, about 20 specimens per hospital were collected per surveillance week. Samples were sent to the Shanghai CDC laboratory for identification of types/subtypes of influenza virus, according to a standard protocol [28]. During the period when the contact survey was conducted (December 2017 to March 2018), A(H1N1)pdm09 was the circulating influenza type in Shanghai. It is important to stress that in China, general practitioners operate inside the hospital structure; thus, the analyzed data contains visits to general practitioners, equivalent to ILI data collected in the United States and Europe. The weekly number of ILI positive cases was defined as the product of the weekly proportion of specimens that tested positive for influenza A(H1N1)pdm09 to obtain a better proxy of influenza activity. Similar to previous studies, we refer to this indicator as ILI^+^ [29]. The estimated weekly number of ILI^+^ were used for the calibration of our modeling analysis. These estimates are not disaggregated by age group, as the ILI data used in this analysis include only weekly totals.

### 2.2 Statistical Analysis

#### 2.2.1 Covariates

Several covariates were included in the analysis to adjust for characteristics and sources of potential influence on human contact patterns. These covariates are age, gender, household size, occupation type, years lived in Shanghai, weekday, and type of day the diary was completed. Occupation type was separated into three categories: student, employed, and not employed. Participants attending preschool were included in the student category, and the retired participants were included in the not employed category. For the regression analysis, age and household size were normalized by calculating the difference from the means within each category of the occupation type. The variable “years lived in Shanghai” was defined as the number of years a participant had been a resident in Shanghai; categories included: < 6 years, 6–10 years, or > 10 years/entire life. The weekday variable included three levels: Monday–Friday, Saturday, and Sunday. Type of day was defined as whether the diary was completed on a regular day, irregular day, or New Year holiday. Regular days were considered as days when participant's activities did not deviate from their normal schedule. The irregular day referred to days when a person had significant variations to their normal day schedules (e.g., day off or school holiday). New Year holiday identified diaries completed on irregular days during the New Year holiday, from January 26 to February 22, 2018. Interview responses indicating a participant did not know or was unwilling to answer were recoded as missing.

In the regression analysis, we also included two meteorological covariates: the seasonal trend and the daily variations in temperature. We estimated the seasonal trend by fitting a cubic smoothing spline to the maximum daily temperatures between December 1, 2017, and May 30, 2018. Daily temperature variation was defined as the difference between the maximum daily temperatures and the seasonal trend. This was done to differentiate the influences of seasonal variations on human behavior throughout the year from the influences of day‐to‐day temperature variations. Due to its high correlation with temperature, absolute humidity was not included as a predictor in the main analysis but a sensitivity analysis where absolute humidity was used instead of temperature was conducted.

#### 2.2.2 Regression Analysis

The effect of seasonal trend and daily temperature variation on total contacts was analyzed using negative binomial regression while adjusting for the following covariates: normalized age, normalized household size, occupation type, gender, weekdays, years lived in Shanghai, and type of day. Diagnostics were performed to assess regression assumptions. Relative difference in the number of contacts were calculated by exponentiating the coefficients and confidence interval from the regression results. Two sensitivity analyses were performed using alternative meteorological variables. All methods remained the same except seasonal trends and daily variations were calculated using average daily temperatures or the daily maximum absolute humidity. We also performed a sensitivity analysis where we assessed the effects of seasonal trends and daily temperature variation on community contacts (i.e., contacts in the settings outside homes, schools, and workplaces) while adjusting for the same covariates (see Supporting Information).

#### 2.2.3 Estimated Number of Contacts

To estimate the average daily number of contacts for each day of the study period, we first created a population of 100,000 individuals by sampling study population with replacement. For each individual in the created sample, covariates of normalized age, normalized household size, occupation type, and years lived in Shanghai were inherited from the study population. Then, we estimated the expected trend in the number of contacts for each individual using the following procedure. For each calendar date during the study period, we assigned the temperature seasonal trend and daily variations in temperature based on the recorded temperature on that day. The day of the week was identified based on the calendar date, and the type of day was considered regular for all dates outside the New Year holiday period. Based on this dataset, we used the regression coefficients to predict the number of contacts for each day of the study period for each individual of the created population. To estimate the average number of contacts at the population level, we estimated the population mean of the predicted number of contacts for each day. To account for the uncertainty in the regression coefficients, we performed bootstrap sampling (1000 draws with replacement) of the coefficients to create prediction intervals. Each coefficient draw was used to predict the number of contacts for the created population for each day. The 95% interquartile range of the resulting vector of predictions were then used as the 95% PrI for each day.

We also performed a sensitivity analysis to assess the differences in contact patterns without considering the effect of the New Year holiday.

### 2.3 Mathematical Modeling Analysis

#### 2.3.1 Influenza Transmission Model

To simulate influenza transmission, we used a traditional homogenous‐mixing SIR model that classified individuals into three compartments: susceptible (*S*), infectious (*I*), and removed (*R*). The model is regulated by the following system of ordinary differential equations:

$$\dot{S}\left(t\right)=-\mathrm{\beta c}\left(t\right)\frac{I\left(t\right)}{N}S\left(t\right)$$

$$\dot{I}\left(t\right)=\mathrm{\beta c}\left(t\right)\frac{I\left(t\right)}{N}S\left(t\right)-\mathrm{\gamma I}\left(t\right)$$

$$\dot{R}\left(t\right)=\mathrm{\gamma I}\left(t\right)$$

where

- N represents the total population of Shanghai (24,860,000 inhabitants as of 2017) [30];
- β represents the per‐contact transmission risk;
- γ represents the inverse of the effective incubation period. According to the literature, for the traditional SIR model [31], γ also corresponds to the inverse of the generation time, which was set at 3.0 days [32];
- $c\left(t\right)$ represents the per‐capita contact rate at time t (where t corresponds to 1 day), which was derived from the analysis of the contact survey data.

The contact‐dependent net reproduction number was then estimated as $R_{t}=\frac{\mathrm{\beta c}\left(t\right)}{\gamma }\frac{S\left(t\right)}{N}$ [33].

It is important to stress that our system (and consequently also the equation for $R_{t}$) assumes that the net reproduction number is proportional to the number of contacts without incorporating additional factors such temperature, absolute humidity, and the type of contact (e.g., indoor vs. outdoor) that may affect influenza transmissibility.

#### 2.3.2 Model Calibration

The influenza transmission model was calibrated on the weekly number of ILI^+^ estimated from the 2017–2018 influenza surveillance data collected in Shanghai (i.e., the same time period and location of the contact survey). We defined the likelihood of observing the reported total number of ILI^+^ given a negative binomial distribution with a mean given by the number of weekly infections estimated by the model multiplied by a reporting rate and a given over‐dispersion. We then used MCMC to explore this likelihood and estimate the joint posterior distributions of the per‐contact transmission risk, the reporting rate, the number of initially infected individuals, and the over‐dispersion of the negative binomial distribution used in the likelihood. Metropolis‐Hastings sampling was utilized to identify candidate parameters for each iteration of the MCMC. The details for implementing the MCMC process can be found in the Supporting Information.

## 3 Results

### 3.1 Contact Patterns

We investigated the seasonality of human contact patterns using data collected through a cross‐sectional diary‐based contact survey of 965 participants [25]. A total of 18,116 close contacts (mean = 18.7 per day per participant, interquartile range [IQR]: 4.0–30.0) were analyzed (Table 1). The number of participants per week ranged from 0 to 157 (Figure S1). The study sample was balanced between female (50.9%) and male (49.1%) participants. Nearly half of the participants were between the ages of 19 and 59 (49.4%) and were employed (41.5%), whereas a majority had lived in Shanghai for more than 10 years or their entire life (89.3%). Additional descriptive statistics of the participants are reported in Table S1. Adults 60 years and older reported 12.6 contacts on average (IQR: 4.0–16.0), whereas those 19–59 years had 21.4 contacts (IQR: 5.0–33.0), and participants 0–18 years old had an average of 20.5 contacts (IQR: 4.0–34.0). Employed persons reported a number of contacts similar to that of students (22.5, IQR: 6.0–34.0, vs. 21.2, IQR: 4.0–34.0, on average). 73.1% of participants completed the diaries during a weekday (Monday–Friday). During weekdays, respondents had more contacts on average (mean: 20.3, IQR: 5.0–32.0) compared to Saturday (mean: 13.8, IQR: 4.0–17.0) and Sunday (mean: 15.4, IQR: 4.0–20.5). Most participants (65.8%) completed their contact diaries on a regular day. Seasonal trend and daily temperature variation were significantly correlated with the New Year holiday due to the New Year occurring during the colder season (Figure S2).

**TABLE 1:** Descriptive statistics of the participants and their total contacts.

		Number of contacts	
	*N* (%)	Mean	IQR
Total	965 (100.0)	18.7	(4.0, 30.0)
Gender			
Female	491 (50.9)	18.5	(4.0, 29.5)
Male	474 (49.1)	19.0	(4.3, 31.0)
Age group			
0–18	221 (22.9)	20.5	(4.0, 34.0)
19–59	477 (49.4)	21.4	(5.0, 33.0)
60+	267 (27.7)	12.6	(4.0, 16.0)
Occupation type			
Student	252 (26.2)	21.2	(4.0, 34.0)
Employed	400 (41.5)	22.5	(6.0, 34.0)
Not employed	307 (31.8)	12.0	(3.0, 15.0)
Missing	6 (0.6)	13.0	(3.0, 9.0)
Years lived in Shanghai			
< 6 years	47 (4.9)	17.7	(4.0, 22.5)
6–10 years	52 (5.4)	20.9	(5.0, 34.5)
> 10 years/entire life	862 (89.3)	18.7	(4.0, 29.8)
Missing	4 (0.4)	13.8	(3.8, 19.5)
Type of day			
Regular day	635 (65.8)	18.7	(4.0, 31.0)
New Year holiday	211 (21.9)	17.7	(5.0, 21.0)
Irregular day	96 (9.9)	22.4	(6.8, 36.0)
Missing	23 (2.4)	14.9	(4.0, 26.0)
Weekday			
Monday–Friday	705 (73.1)	20.3	(5.0, 32.0)
Sunday	143 (14.8)	15.4	(4.0, 20.5)
Saturday	117 (12.1)	13.8	(4.0, 17.0)

Abbreviation: IQR = interquartile range.

### 3.2 Seasonality of Contact Patterns

**FIGURE 1:**
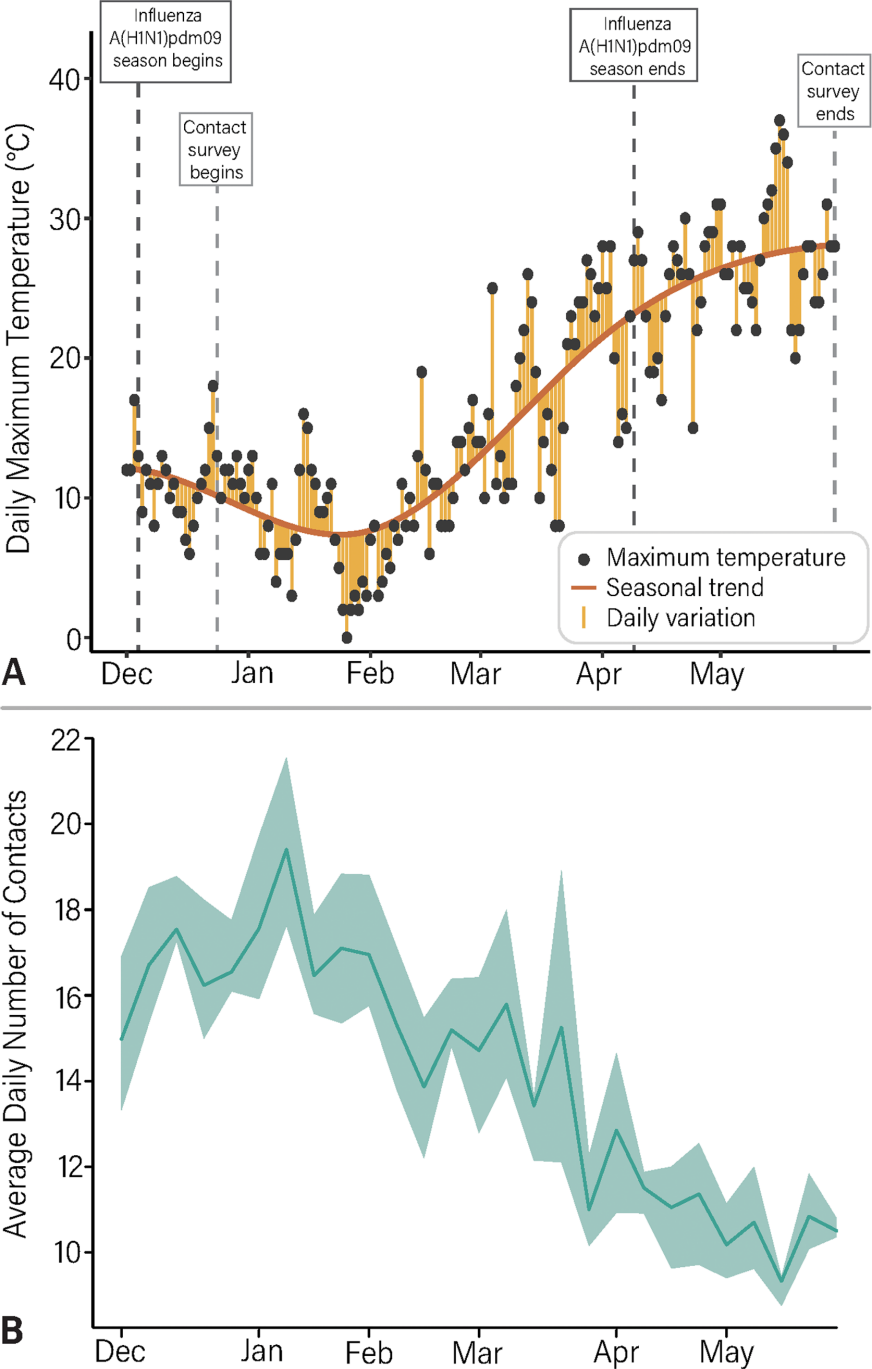
(A) Daily maximum temperature (°C) for each day from December 1, 2017, to May 30, 2018, the seasonal trend of the temperatures, daily variation between the maximum temperature, and seasonal trend. (B) Estimated daily number of total contacts for each week from December 1, 2017, to May 30, 2018. The line and shaded area represent the mean and 95% PrI of the mean daily values for each season, respectively.

We found that both the estimated seasonal trend and daily variation in temperatures (Figure 1A) were associated with a significant decrease in the number of contacts (Table 2 and Figure S3). Specifically, for each degree increase in seasonal trend, the difference in the logs of the expected number of contacts decreases by 0.013 (*p* = 0.003; 95% CI: −0.022, −0.004). In other words, for an increase of 1°C, the number of contacts is expected to decrease by 1.3%. Likewise, for each degree of daily variation in temperature, the difference in the logs of the expected number contacts decreases by 0.019 (*p* = 0.009, 95% CI: −0.034, −0.004); that is, the number of contacts is expected to decrease by 1.9%. We estimated an average of 16.4 (95% PrI: 15.1–17.5) contacts per day in December 2017 that increased to an average of 17.6 contacts (95% PrI: 16.5–19.3) in January 2018 and declined to an average of 10.3 (95% PrI: 9.4–10.8) in May 2018 (Figure 1B and Figure S4). We found consistent results in a sensitivity analysis where we did not consider the New Year holiday period (Figure S5).

**TABLE 2:** Negative binomial regression models of the effects of seasonal trend and daily temperature variation on total contacts adjusting for the covariates.

Variable	Number of contacts		
	Coefficient	*p*	95% CI
Seasonal trend	−0.013	0.003**	(−0.022, −0.004)
Daily temperature variation	−0.019	0.009**	(−0.034, −0.004)
Normalized age	0.005	0.114	(−0.001, 0.011)
Normalized household size	0.059	0.054	(−0.002, 0.121)
Occupation type			
Employed	Ref		
Not employed	−0.680	< 0.0001***	(−0.834, −0.525)
Students	−0.010	0.198	(−0.253, 0.055)
Years lived in Shanghai			
> 10 years/entire life	Ref		
< 6 years	−0.177	0.224	(−0.459, 0.123)
6–10 years	0.054	0.694	(−0.209, 0.334)
Weekday			
Monday–Friday	Ref		
Saturday	−0.395	< 0.0001***	(−0.584, −0.199)
Sunday	−0.202	0.024*	(−0.378, −0.021)
Type of day			
Regular day	Ref		
Irregular day	0.162	0.121	(−0.039, 0.372)
New Year holiday	−0.183	0.056	(−0.372, 0.007)
Gender			
Male	Ref		
Female	0.060	0.344	(−0.067, 0.187)

*Note:* Total *N* = 965. *df* = 910.

Abbreviation: CI = confidence intervals.

* *p* < 0.05,

** *p* < 0.01, and

*** *p* < 0.001.

Our primary analysis utilized maximum daily temperature to gauge seasonal trends and daily variations in temperature. To evaluate the robustness of our findings, we conducted two sensitivity analyses employing two alternative meteorological measurements: average daily temperature and absolute humidity. Regression results based on these two alternative measurements yield consistent results (see Table S2, Table S3, and Figure S6). The estimated coefficient for the seasonal trend was −0.016 (95% CI: −0.026, −0.006) when using the average daily temperature and −0.014 (95% CI: −0.024, −0.003) when using the absolute humidity; the estimated coefficient for the daily variation was −0.025 (95% CI: −0.045, −0.005) when using the average daily temperature and −0.033 (95% CI: −0.049, −0.016) when using the absolute humidity.

We conducted an additional analysis to assess the relationship between seasonality and contact occurring in community settings (i.e., any setting excluding home, work, and school). The number of community contacts by participant characteristics is reported in Table S4. We found that the difference in the logs of expected contacts significantly decreased by 0.025 (*p* = 0.003, 95% CI: −0.048 to −0.013) for each degree increase in seasonal trend (i.e., the number of contacts is expected to decrease by 2.5%), which was nearly double the effect we estimated for all contacts (Table S5 and Figure S7). This supports the idea that contacts in the community are those most influenced by weather, whereas contacts at home, school, and work tend to follow more consistent patterns.

### 3.3 Seasonality of Influenza

**FIGURE 2:**
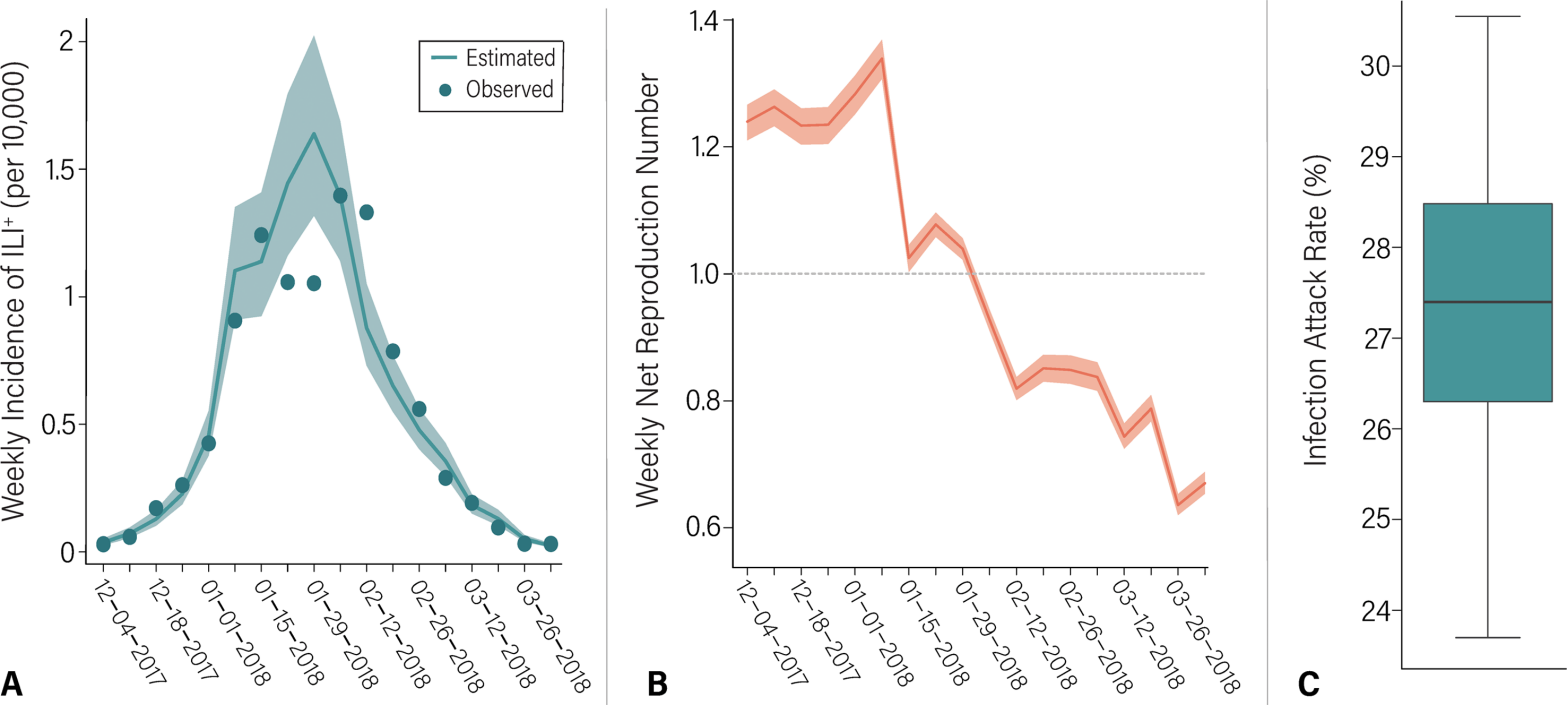
(A) Estimated weekly incidence of ILI^+^ infections during the 2017–2018 influenza season in Shanghai, China. Dots represent the observed data; line and shaded area represent the mean and 95% CI of model simulations. ILI^+^ refers to the estimated weekly number of ILI cases that tested positive for influenza A(H1N1)pdm09. (B) Estimated posterior distribution of weekly net reproduction number. The line and shaded area represent the mean and 95% CI, respectively. (C) Posterior distribution of the final infection attack rate for the 2017–2018 influenza season. The boxplot reports quantiles 0.025, 0.25, 0.5, 0.75, and 0.975 of the distribution.

After adjusting for the reporting rate (Figure S8), the mean number of ILI^+^ estimated by the mathematical model based on the predicted contact patterns is consistent with the reported data (Figure 2A). However, our model slightly overestimated the mean number of ILI^+^ during the New Year holiday, which may be due to a lower reporting rate of ILI during the holiday season, as observed in other countries [34]. The net reproduction number, which compounds the effects of preexisting immunity (e.g., due to vaccination or previous infection), changes in contact patterns and behaviors, and the transmissibility of the virus, was estimated to increase from 1.24 (95% CI: 1.21–1.27) in December to a peak of 1.34 (95% CI: 1.31–1.37) in January. Then, the epidemic started to enter a decline phase in early February when the net reproduction number estimated by the model fell below the epidemic threshold (Figure 2B). The model also provides estimates of the median infection attack rate, which was estimated to reach 27.4% (95% CI: 23.7–30.5%) by the end of the season (Figure 2C).

## 4 Discussion

Our study leverages contact patterns and epidemiological data simultaneously collected in the same study site when social distancing measures were not in place. We found a significant association between the number of contacts and both seasonal and day‐to‐day changes in temperature. Furthermore, the influenza transmission model, informed by this association, captured the temporal dynamics of influenza during the analyzed season, suggesting that temperature‐related variations in contacts are a potential contributor to the seasonality of influenza. In addition to reproducing the temporal dynamics of the 2017–2018 influenza season, our modeling analysis also provides estimates of the net reproduction number and infection attack rate, which are comparable with previous literature estimates [35, 36, 37, 38], supporting the robustness of our results. Moreover, our estimated net reproduction number showed a non‐monotonous trend by increasing through early January before declining through the rest of the influenza season.

Our modeling analysis considered a direct relationship between seasonal contact patterns without any additional factors. For example, the risk of SARS‐CoV‐2 transmission differs between indoor and outdoor contacts [39], which may also apply to influenza. However, our analysis did not make this distinction due to the absence of such information in the contact survey. Moreover, the seasonal trend in influenza transmission may be attributed to other factors, but it is possible that they synergistically contribute to the observed seasonal trends in influenza infections. It is also important to recognize that our results are based on the analysis of one influenza A(H1N1)pdm09 season in Shanghai—as that was the influenza virus circulating during the study period. Thus, it is unclear to what extent our results are applicable to other influenza viruses and locations. Additional studies examining the intricate relationship between weather and climatic conditions, human contact patterns, intervention measures, and the biological characteristics of pathogens are warranted.

This study has several limitations. First, we have only 6 months of contact data, and it would be important to collect year‐round data to draw more definitive conclusions on the relationship between the total number of contacts and the seasonal trend and daily temperature variation for the entire year. Second, our analysis is based on a homogenous‐mixing compartmental model. The addition of age‐dependent factors to the model (e.g., age‐specific contact patterns, transmission risks, and vaccination coverages) could allow for obtaining a deeper understanding of influenza transmission patters and epidemiology. An age‐dependent model would, however, require validation against epidemiological data disaggregated by age, which is not available to us. Third, we do not explicitly model vaccination. However, on the one hand, its effectiveness in protecting against infection is accounted for in the model by the estimated per‐contact transmission risk. On the other hand, because our study does not aim at providing estimates of influenza burden (for instance, in terms of number of hospitalizations and deaths), the model does not need to consider vaccine effectiveness in protecting against severe clinical outcomes.

Despite these limitations, our study can serve as a steppingstone for future research exploring the association between seasonality, human contact patterns, and the epidemiology of respiratory infectious diseases. Understanding their relationships may be especially important in the post‐pandemic period, when COVIDD‐19 may start following more marked seasonal trends, possibly aligning with those of influenza and/or RSV. Such a possible synergy may put more strain on healthcare systems around the world.

In conclusion, our analysis highlights the interaction between the seasonal fluctuations in contact patterns and epidemic dynamics. The mechanism proposed in this study could improve our understanding of the transmission patterns of respiratory pathogens and help elucidate their seasonal trends.

## Author Contributions

**Allisandra G. Kummer:** formal analysis, writing–original draft. **Juanjuan Zhang:** formal analysis, writing–review and editing. **Chenyan Jiang:** formal analysis, writing–review and editing. **Maria Litvinova:** methodology, writing–review and editing. **Paulo C. Ventura:** methodology, writing–review and editing. **Marc A. Garcia:** writing–review and editing. **Alessandro Vespignani:** writing–review and editing. **Huanyu Wu:** supervision, writing–review and editing. **Hongjie Yu:** conceptualization, methodology, supervision, writing–review and editing. **Marco Ajelli:** conceptualization, methodology, supervision, writing–original draft.

## Conflicts of Interest

H.Y. has received research funding from Sanofi Pasteur, GlaxoSmithKline, Yichang HEC Changjiang Pharmaceutical Company, Shanghai Roche Pharmaceutical Company, and SINOVAC Biotech Ltd. M.A. has received research funding from Seqirus. None of this funding is related to this research. The other authors declare no conflicts of interest.

### Peer Review

The peer review history for this article is available at https://www.webofscience.com/api/gateway/wos/peer‐review/10.1111/irv.13301.

## Supporting information


**Figure S1.** Number of total contacts and number of participants interviewed in Shanghai, China by week from December 24, 2017, to May 30, 2018.
**Table S1.** Descriptive statistics for the variables not reported in the main text.
**Figure S2.** Correlation matrix of the independent variables chosen for the main analysis. The * denotes a statistically significant correlation.
**Figure S3.** Incidence rate ratios of seasonal trend, daily temperature variation, and the covariates of interest for the total contacts.
**Figure S4.** Estimated daily number of contacts for each day when seasonal trend, daily variation, and weekday vary while all other variables were fixed between November 27, 2017, and May 30, 2018. The line and shaded area represent the mean and 95% PrI of the daily values, respectively.
**Figure S5.** Estimated daily number of contacts for each week when seasonal trend, daily variation, and weekday vary while all other variables were fixed between December 1, 2017, to May 30, 2018. The line and shaded area represent the mean and 95% PrI of the weekly values, respectively.
**Table S2.** Negative binomial regression model of the effects of the seasonal trend and daily temperature variation using the average daily temperature on total contacts adjusting for the covariates of interest.
**Table S3.** Negative binomial regression model of the effects of seasonal trend and daily temperature variation calculated using absolute humidity on total contacts adjusting for the covariates of interest.
**Figure S6.** Estimated average daily number of contacts for each week when seasonal trend, and daily variation vary with an adjustment for Sundays while all other variables are fixed. The line and shaded area represent the mean and quantiles 0.025 and 0.975 of the daily values. **A.** Maximum temperature. **B.** Mean temperature. **C.** Absolute humidity.
**Table S4.** Descriptive Statistics for the community contacts by participants’ gender, age, occupation, length of time living in Shanghai, and type of day and weekday of participation.
**Table S5.** Negative binomial regression model of the effects of seasonal trend and daily temperature variation on community contacts adjusting for the covariates of interest.
**Figure S7.** Incidence rate ratios of seasonal trend, daily temperature variation, and the covariates of interest for the community contacts.
**Figure S8.** Trace plots of the estimated model parameters.

## Data Availability

Data and code to replicate the results are available on GitHub: https://github.com/CEPH‐Lab/influenza‐seasonality‐shanghai.
