# Supplementary material for: Evaluating Seasonal Variations in Human Contact Patterns and Their Impact on the Transmission of Respiratory Infectious Diseases
Source: Influenza Other Respir Viruses. 2024 May 11;18(5):e13301. doi: 10.1111/irv.13301 (PMC11087848; doi:10.1111/irv.13301)
Supplement: Supplementary file 1 — Figure S1. Number of total contacts and number of participants interviewed in Shanghai, China by week from December 24, 2017, to May 30, 2018. Table S1. Descriptive statistics for the variables not reported in the main text. Figure S2. Correlation matrix of the independent variables chosen for the main analysis. The * denotes a statistically significant correlation. Figure S3. Incidence rate ratios of seasonal trend, daily temperature variation, and the covariates of interest for the total contacts. Figure S4. Estimated daily number of contacts for each day when seasonal trend, daily variation, and weekday vary while all other variables were fixed between November 27, 2017, and May 30, 2018. The line and shaded area represent the mean and 95% PrI of the daily values, respectively. Figure S5. Estimated daily number of contacts for each week when seasonal trend, daily variation, and weekday vary while all other variables were fixed between December 1, 2017, to May 30, 2018. The line and shaded area represent the mean and 95% PrI of the weekly values, respectively. Table S2. Negative binomial regression model of the effects of the seasonal trend and daily temperature variation using the average daily temperature on total contacts adjusting for the covariates of interest. Table S3. Negative binomial regression model of the effects of seasonal trend and daily temperature variation calculated using absolute humidity on total contacts adjusting for the covariates of interest. Figure S6. Estimated average daily number of contacts for each week when seasonal trend, and daily variation vary with an adjustment for Sundays while all other variables are fixed. The line and shaded area represent the mean and quantiles 0.025 and 0.975 of the daily values. A. Maximum temperature. B. Mean temperature. C. Absolute humidity. Table S4. Descriptive Statistics for the community contacts by participants’ gender, age, occupation, length of time living in Shanghai, and type of day and weekd [file IRV-18-e13301-s001.pdf]

Evaluating seasonal variations in human contact patterns and their impact on the transmission of respiratory infectious diseases

Supplementary Material

Allisandra G. Kummer[1,†], Juanjuan Zhang[2,6,†], Chenyan Jiang[3,†], Maria Litvinova[1], Paulo C. Ventura[1], Marc A. Garcia[4], Alessandro Vespignani[5], Huanyu Wu[3,‡,*], Hongjie Yu[2,6‡,*], Marco Ajelli[1,‡,*]

1. Laboratory for Computational Epidemiology and Public Health, Department of Epidemiology and Biostatistics, Indiana University School of Public Health, Bloomington, IN, USA
2. Shanghai Institute of Infectious Disease and Biosecurity, Department of Epidemiology, School of Public Health, Fudan University, Shanghai, China.
3. Shanghai Municipal Center for Disease Control and Prevention, Shanghai, China
4. Lerner Center for Public Health Promotion, Aging Studies Institute, Department of Sociology, and Maxwell School of Citizenship & Public Affairs, Syracuse University, Syracuse, NY, USA
5. Laboratory for the Modeling of Biological and Socio-technical Systems, Northeastern University, Boston, MA, USA
6. Department of Epidemiology, School of Public Health, Fudan University, Key Laboratory of Public Health Safety, Ministry of Education, Shanghai, China

[†]Co-first author
[‡]Co-senior author
*Corresponding authors

Seasonal Trend and Contact Patterns *3*

Main Analysis **3**
Descriptive Analysis 3
Correlation Analysis 4
Regression Analysis and Incidence Rate Ratios 4

Estimated Contacts **5**
Daily Number of Contacts 5
Additional Analyses: Holidays 5

Sensitivity Analysis: Additional Meteorological Measurements **7**
Mean Temperature 7
Absolute Humidity 8
Estimated Number of Contacts 9

Analysis of Community Contacts **9**
Descriptive Analysis 9
Regression Analysis 11
Incidence Rate Ratio 11
Estimated Number of Community Contacts 12

Modeling Influenza Spread *13*

Weekly Number of ILI$^+$ in Shanghai, China **13**

Influenza Transmission Model **13**
Model Calibration 13

Seasonal Trend and Contact Patterns

Main Analysis

Descriptive Analysis

The number of participants per week ranged from 0 to 157, and the total number of contacts each week ranged from 0 to 3,331 contacts (Fig. S1).

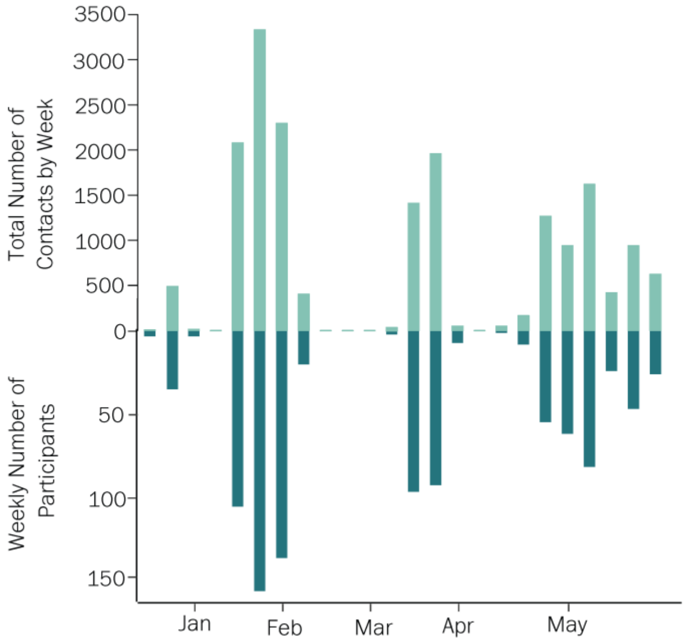

Figure S1. Number of total contacts and number of participants interviewed in Shanghai, China by week from December 24, 2017, to May 30, 2018.

Age was defined as the age of the participant on the calendar date they completed the contact diary. Household size was defined as the number of individuals living in the same house as the participant. The average age of participants was 40.7 years old (IQR: 21.0 – 60.0), the average household size was approximately 2 people, not including the participant, and the average maximum temperature in Shanghai during the study period was 15.1 °C (IQR: 7.0 – 25.0) (Tab. S1). For the main analysis, age and household size were normalized by calculating their overall means for each category of the occupation type. These means were then subtracted from the age or household size of participants in that category to determine their normalized age and their normalized household size.

Table S1. Descriptive statistics for the variables not reported in the main text.

	Mean	IQR
**Age**	40.7	(21.0, 60.0)
**Household size**[a]	2.1	(1.0, 7.0)
**Maximum Temperature**	15.1	(7.0, 25.0)

Note. total *N* = 965.
[a]Household size does not include the participant.

Correlation Analysis

Collinearities between independent variables were assessed through a Spearman correlation analysis and a Chi-square test (Fig. S2). The seasonal trend and daily temperature variations were inversely connected with the regularity of the daily schedules, reflecting the coinciding of the New Year holidays with the colder season. The rest of the statistically significant correlations were small in size.

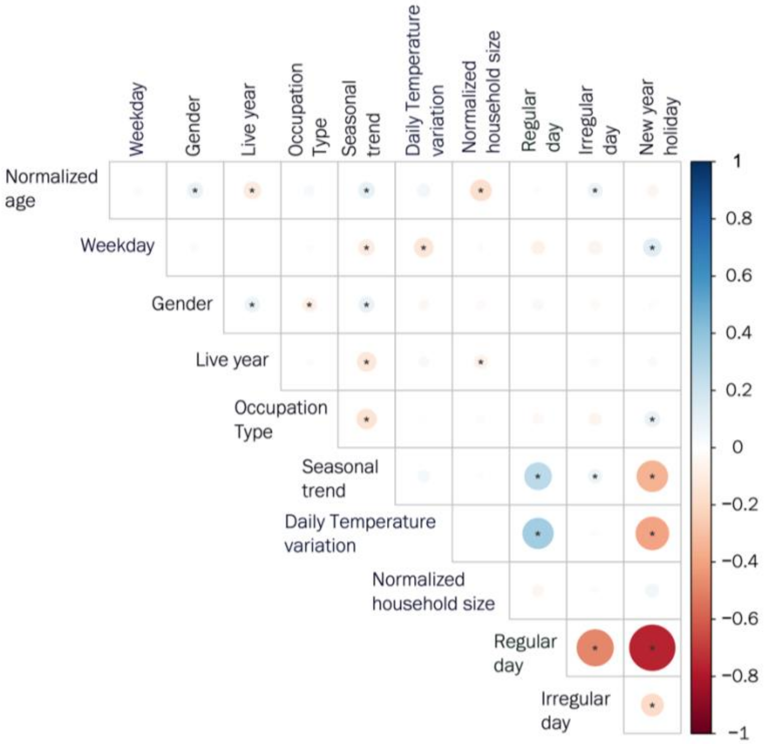

Figure S2. Correlation matrix of the independent variables chosen for the main analysis. The * denotes a statistically significant correlation.

Regression Analysis and Incidence Rate Ratios

We considered variation in school closure in the regression analysis by extending the holiday period to begin on January 22, 2018. This date was chosen based on the median number of college students whose holiday break began before Shanghai's public school system. We also assessed the regression analysis for outliers. An outlier was defined as having a Cook's Distance 20 times greater than the mean value. Four observations met the criteria for an outlier; therefore, they were excluded from the final model.

Incidence rate ratios (IRR) were calculated by exponentiating the coefficients and confidence interval from the regression results (Fig. S3). Contact rates were lower on Sundays (IRR = 0.817, 95% CI: 0.685, 0.979) and Saturdays (IRR = 0.674, 95% CI: 0.558, 0.820) compared to Monday through Friday. Not employed participants had lower rates of contact than those employed (IRR = 0.507, 95% CI: 0.434, 0.592). As seasonal trends moved from colder to warmer temperatures, the rate of contacts decreased (IRR = 0.987, 95% CI: 0.978, 0.996). Likewise, increased variations in daily temperatures showed decreased rates in total contacts (IRR = 0.981, 95% CI: 0.966, 0.996).

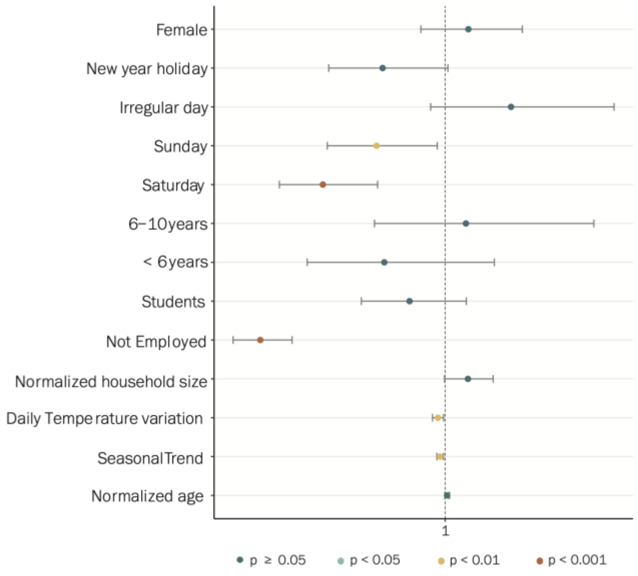

Figure S3. Incidence rate ratios of seasonal trend, daily temperature variation, and the covariates of interest for the total contacts.

Estimated Contacts

Daily Number of Contacts

We estimated the number of contacts for each day from December 1, 2017, to May 30, 2018, using the results of the negative binomial regression. By looking at the daily estimates (instead of grouped by week, as reported in the main text), the effect of weekends on the number of contacts is clearly visible (Fig. S4).

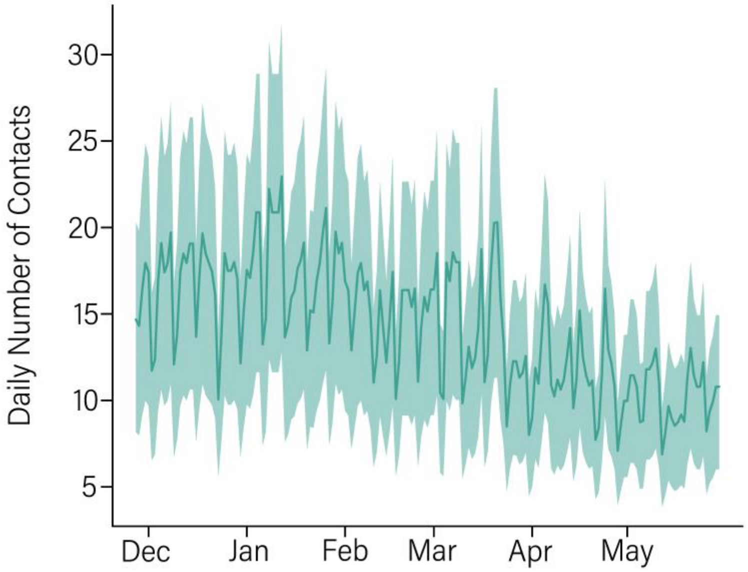

Figure S4. Estimated daily number of contacts for each day when seasonal trend, daily variation, and weekday vary while all other variables were fixed between November 27, 2017, and May 30, 2018. The line and shaded area represent the mean and 95% PrI of the daily values, respectively.

Additional Analyses: Holidays

We performed an additional analysis for estimating the number of contacts without considering the New Year holiday period. Without consideration for the New Year holiday (Fig. S5), we see a higher number of contacts during the holiday break period compared to the main analysis with a later peak at the end of January (20.5 contacts, 95% PrI 18.4-22.6).

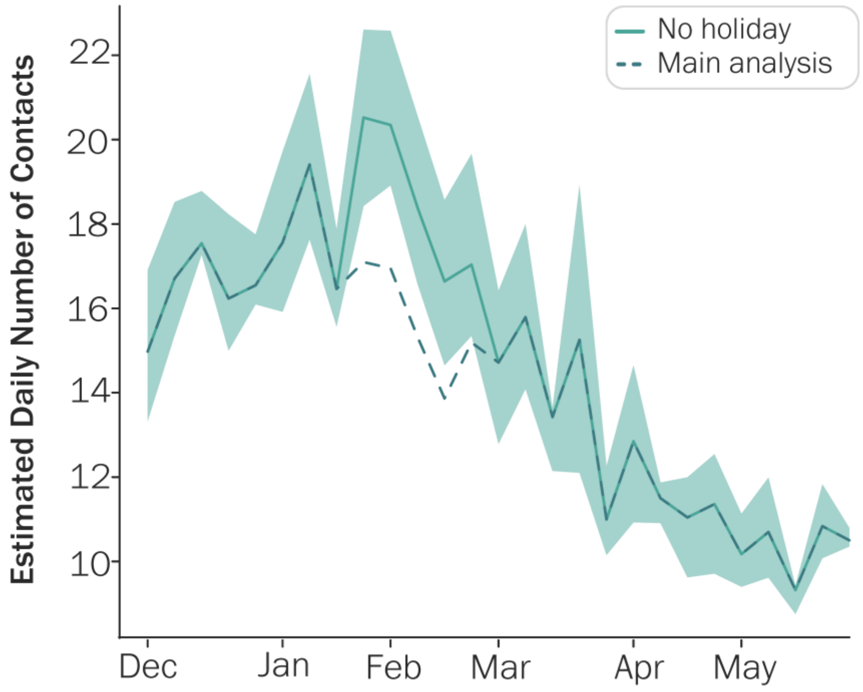

Figure S5. Estimated daily number of contacts for each week when seasonal trend, daily variation, and weekday vary while all other variables were fixed between December 1, 2017, to May 30, 2018. The line and shaded area represent the mean and 95% PrI of the weekly values, respectively.

Sensitivity Analysis: Additional Meteorological Measurements

Mean Temperature

In the main analysis, we considered maximum daily temperature as most contacts take place during the daytime, when the temperature is highest. Here, we repeat the analysis by considering mean daily temperatures. When the seasonal trend is calculated with respect for mean temperature, not-employed persons, Saturdays, Sundays, and new year holidays showed significant decreases in expected contacts compared to employed individuals, Mondays-Fridays, and regular days (Tab. S2). Seasonality showed significant decreases in the number of total contacts. Specifically, for each degree increase in the seasonal trend, the difference in the logs of expected contacts decreases by 0.016 ($p = 0.002$, 95% Confidence Interval (CI): -0.026, -0.006). Likewise, the difference in the logs of expected total contacts decreases by 0.025 for each degree of daily temperature variation ($p = 0.011$, 95% CI: -0.045, -0.005). These results confirm those obtained for the main analysis.

Table S2. Negative binomial regression model of the effects of the seasonal trend and daily temperature variation using the average daily temperature on total contacts adjusting for the covariates of interest.

Variable	**Total Contacts**		
	Coefficient	p-value	95% CI
**Seasonal trend**	-0.016	0.002**	(-0.026, -0.006)
**Daily Temperature variation**	-0.025	0.011*	(-0.045, -0.005)
**Normalized age**	0.005	0.106	(-0.001, 0.012)
**Normalized household size**	0.058	0.057	(-0.002, 0.120)
**Occupation Type**			
Employed	ref		
Not Employed	-0.677	0.000***	(-0.832, -0.522)
Students	-0.102	0.191	(-0.255, 0.053)
**Years lived in Shanghai**			
>10 years/entire life	ref		
< 6 years	-0.169	0.245	(-0.451, 0.131)
6-10 years	0.060	0.663	(-0.204, 0.341)
**Weekday**			
Monday – Friday	ref		
Saturday	-0.375	0.000***	(-0.565, -0.178)
Sunday	-0.192	0.033*	(-0.369, -0.010)
**Type of Day**			
Regular day	ref		
Irregular day	0.165	0.115	(-0.037, 0.375)
New year holiday	-0.210	0.039*	(-0.411, -0.008)
**Gender**			
Male	ref		
Female	0.062	0.334	(-0.066, 0.189)

Note. total $N = 965$. $df = 910$. CI =confidence intervals.
*p < 0.05. ** p < 0.01. ***p < 0.001.

Absolute Humidity

When seasonal trend is calculated for absolute humidity, not-employed persons, Saturdays, Sundays, and new year holidays showed significant decreases in expected contacts compared to employed individuals, Mondays-Fridays, and regular days (Tab. S3). Seasonality showed significant decreases in the number of total contacts. Specifically, for each degree increase in the seasonal trend, the difference in the logs of expected contacts decreases by 0.014 (p = 0.010, 95% Confidence Interval (CI): -0.024, -0.003). Likewise, expected total contacts decreases by 0.033 for each degree of daily temperature variation (p = 0.000, 95% CI: -0.049, -0.016). These results confirm those obtained for the main analysis.

Table S3. Negative binomial regression model of the effects of seasonal trend and daily temperature variation calculated using absolute humidity on total contacts adjusting for the covariates of interest.

Variable	Total Contacts		
	Coefficient	p-value	95% CI
**Seasonal trend**	-0.014	0.010*	(-0.024, -0.003)
**Daily Temperature variation**	-0.033	0.000***	(-0.049, -0.016)
**Normalized age**	0.006	0.088	(-0.001, 0.012)
**Normalized household size**	0.053	0.081	(-0.007, 0.115)
**Occupation Type**			
Employed	ref		
Not Employed	-0.665	0.000***	(-0.818, -0.511)
Students	-0.091	0.237	(-0.244, 0.063)
**Years lived in Shanghai**			
>10 years/entire life	ref		
< 6 years	-0.167	0.249	(-0.449, 0.132)
6-10 years	0.075	0.586	(-0.188, 0.354)
**Weekday**			
Monday – Friday	ref		
Saturday	-0.387	0.000***	(-0.575, -0.191)
Sunday	-0.168	0.062	(-0.344, 0.014)
**Type of Day**			
Regular day	ref		
Irregular day	0.152	0.145	(-0.049, 0.361)
New year holiday	-0.155	0.085	(-0.332, 0.023)
**Gender**			
Male	ref		
Female	0.060	0.342	(-0.067, 0.187)

Note. total *N* = 965. *df* = 910. CI =confidence intervals.
*p < 0.05. ** p < 0.01. ***p < 0.001.

Estimated Number of Contacts

There is a clear seasonal trend in the weekly number of contacts for mean daily temperature (Fig. S7*B*) and absolute humidity (Fig. S7*C*), reflecting trends seen when using maximum daily temperature (Fig. S7*A*).

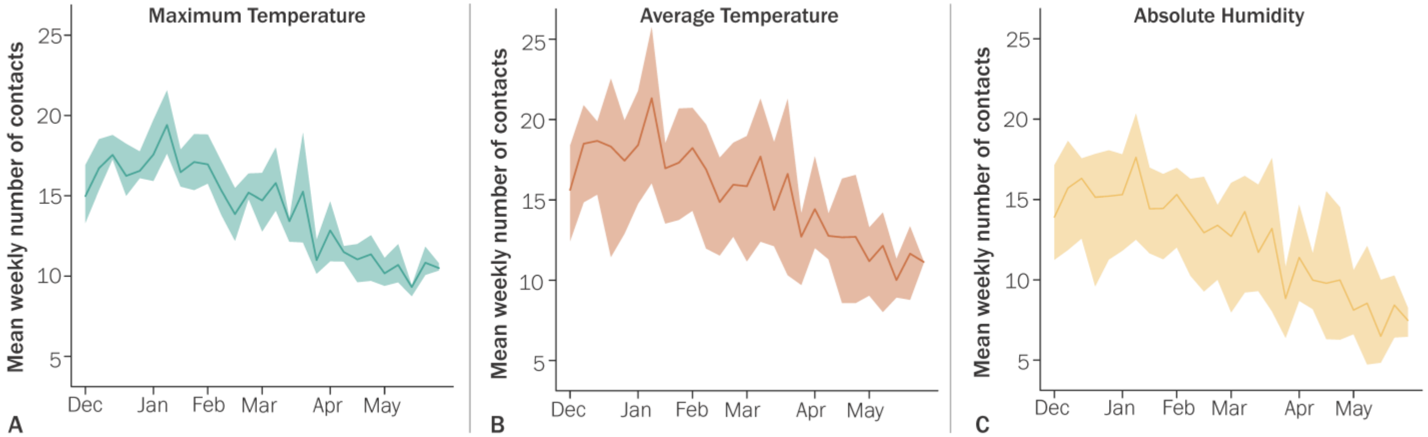

Figure S6. Estimated average daily number of contacts for each week when seasonal trend, and daily variation vary with an adjustment for Sundays while all other variables are fixed. The line and shaded area represent the mean and quantiles 0.025 and 0.975 of the daily values. **A.** Maximum temperature. **B.** Mean temperature. **C.** Absolute humidity.

Analysis of Community Contacts

Descriptive Analysis

Descriptive statistics for the covariates and community contacts are shown in Table S4. Adults 60 years and older reported 8.09 community contacts on average (IQR: 1.0-9.0) whereas those 19-59 years had 5.02 community contacts (IQR: 0.0-3.0) and participants 0-18 years old had an average of 5.23 community contacts (IQR: 0.0-4.0). Employed persons reported a number of community contacts similar to that of students (4.69 vs. 5.13 on average). Participants completing the diaries on Sunday had the most community contacts on average (μ = 11.7, IQR: 0.0-14.0) followed by Saturday with 8.65 community contacts (IQR: 0.0-9.0) compared to the working days. Most contacts were had by persons completing the diary on an irregular day (μ = 8.54, IQR: 0.0-11.0) compared to all other types of days.

Table S4. Descriptive Statistics for the community contacts by participants' gender, age, occupation, length of time living in Shanghai, and type of day and weekday of participation.

	Community Contacts		
	N (%)	Mean	IQR
**Total**	965 (100.0)	5.9	(0.0, 5.0)
**Gender**			
Female	491 (50.9)	6.5	(0.0, 6.0)
Male	474 (49.1)	5.3	(0.0, 5.0)
**Age group**			
0-18	221 (22.9)	5.2	(0.0, 3.0)
19-59	477 (49.4)	5.0	(0.0, 4.0)
60+	267 (27.7)	8.1	(1.0, 9.0)
**Occupation Type**			
Students	252 (26.2)	5.1	(0.0, 3.0)
Employed	400 (41.5)	4.7	(0.0, 3.0)
Not Employed	307 (31.8)	8.3	(1.0, 9.0)
Missing	6 (0.6)	1.5	(1.0, 2.0)
**Years lived in Shanghai**			
< 6 years	47 (4.9)	6.4	(0.0, 5.0)
6-10 years	52 (5.4)	5.4	(0.0, 4.0)
>10 years/entire life	862 (89.3)	5.9	(0.0, 5.0)
Missing	4 (0.4)	1.8	(0.8, 2.0)
**Type of Day**			
Regular day	635 (65.8)	5.1	(0.0, 4.0)
New year holiday	211(21.9)	7.0	(0.0, 6.5)
Irregular day	96 (9.9)	8.5	(0.0, 11.0)
Missing	23 (2.4)	7.0	(3.0, 8.0)
**Weekday**			
Monday - Friday	705 (73.1)	4.3	(0.0, 4.0)
Sunday	143 (14.8)	11.7	(0.0, 14.0)
Saturday	117 (12.1)	8.7	(0.0, 9.0)

Note. IQR = Interquartile range.

Regression Analysis

Normalized age, not-employed persons, Saturdays, Sundays, irregular days, and new year holidays showed significant increases in expected community contacts compared to Employed individuals, working days, and regular days (Tab. S5). For each degree increase in the seasonal trend, the difference in the logs of expected community contacts decreases by 0.025 (p = 0.003, 95% Confidence Interval (CI): -0.048, -0.013).

Table S5. Negative binomial regression model of the effects of seasonal trend and daily temperature variation on community contacts adjusting for the covariates of interest.

Variable	Community Contacts		
	Coefficient	p-value	95% CI
**Seasonal trend**	-0.025	0.003**	(-0.048, 0.013)
**Daily Temperature variation**	-0.017	0.235	(0.006, 0.030)
**Normalized age**	0.018	0.004**	(-0.043, -0.008)
**Normalized household size**	0.012	0.843	(-0.101, 0.127)
**Occupation Type**			
Employed	ref		
Not Employed	0.921	0.0001***	(0.612, 1.232)
Students	-0.093	0.541	(-0.424, 0.243)
**Years lived in Shanghai**			
>10 years/entire life	ref		
< 6 years	0.095	0.734	(-0.439, 0.695)
6-10 years	-0.161	0.547	(-0.666, 0.404)
**Weekday**			
Monday – Friday	ref		
Saturday	1.083	0.0001***	(0.720, 1.470)
Sunday	1.611	0.0001***	(1.257, 1.983)
**Type of Day**			
Regular day	ref		
Irregular day	0.482	0.016*	(0.104, 0.891)
New year holiday	0.505	0.006**	(0.139, 0.875)
**Gender**			
Male	ref		
Female	0.162	0.187	(-0.085, 0.408)

Note. total *N* = 965. *df* = 910. CI =confidence intervals.
*p < 0.05. ** p < 0.01. ***p < 0.001.

Incidence Rate Ratio

Incidence rate ratios (IRR) were calculated by exponentiating the coefficients and confidence interval from the regression results (Fig. S8). Community contact rates were higher on Sundays (IRR = 5.006, 95% PrI: 3.515-7.263) and Saturdays (IRR = 2.953, 95% PrI: 2.055-4.350) compared to Monday through Friday. Not-employed participants had higher rates of community contacts than those who were employed (IRR = 2.512, 95% PrI: 1.844-3.428). As seasonal trends moved from colder to warmer temperatures, the rate of community contacts decreased (IRR = 0.975, 95% PrI: 0.958-0.992).

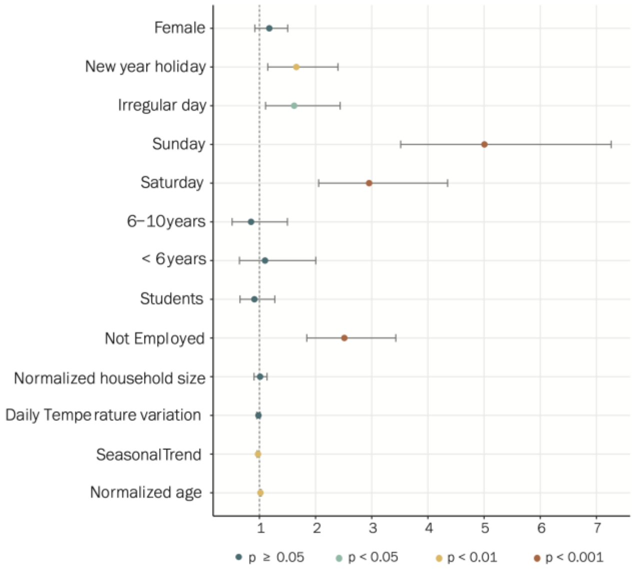

Figure S7. Incidence rate ratios of seasonal trend, daily temperature variation, and the covariates of interest for the community contacts.

Estimated Number of Community Contacts

There is a slightly decreasing trend in the number of community contacts as daily maximum temperature increases which is heavily influenced by the weekend days (Fig. S9). This indicates both a movement from activities in proximity with others (i.e., indoor aggregations) to activities with lower contacts (i.e., outdoor activities) as well as a distinct difference in weekday vs. weekend contacts.

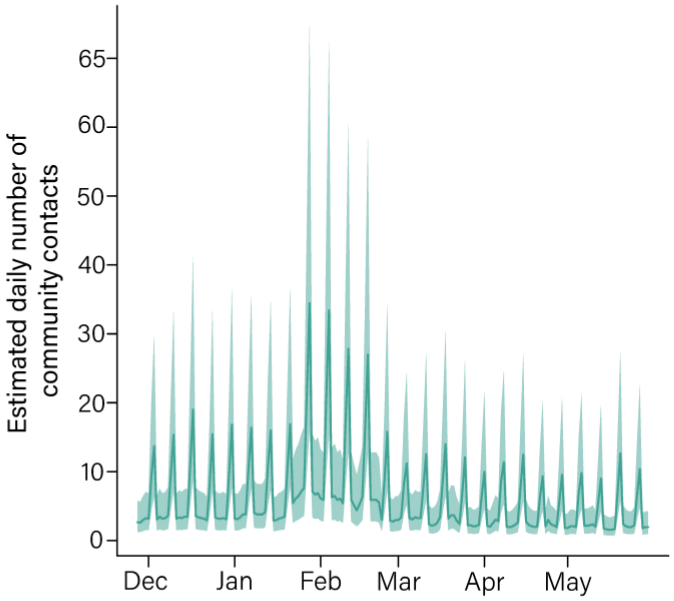

Figure S8. Estimated daily number of community contacts at the population level accounting for variations in the seasonal trend and weekdays. The line and shaded area represent the mean and 95% PrI of the daily values, respectively.

Modeling Influenza Spread

Weekly Number of ILI⁺ in Shanghai, China

The following equation was used to approximate the weekly number of ILI^+ (N_{ILI+}) during the 2017-2018 influenza season.

$$N_{ILI+} = N_{III} * \frac{N_{A(H1N1)pdm09}}{N_{tested}}$$

Where:

- N_{ILI} refers to the weekly number of reported ILI cases;
- $N_{A(H1N1)pdm09}$ refers to the weekly number of ILI cases that tested positive for A(H1N1)pdm09;
- N_{tested} refers to the weekly number of tested ILI cases.

Influenza Transmission Model

We first implemented an infection transmission model that incorporates mean total contacts over time (c_t) estimated for the number of contacts predicted by the regression model. The following equations were used to simulate the transmission process:

$$\dot{S}(t) = -\beta c(t)\frac{I(t)}{N}S(t)$$

$$\dot{I}(t) = \beta c(t)\frac{I(t)}{N}S(t) - \gamma I(t)$$

$$\dot{R}(t) = \gamma I(t)$$

$$\dot{C_t} = \beta c(t)\frac{I(t)}{N}S(t)$$

Where N represents the total population of Shanghai and is set to 24,860,000 which is the population of Shanghai in 2017. The recovery rate corresponds to the inverse of the generation time, which was set to 3.0 days. The contact rate was derived from the analysis of the survey contact data that accounted for the extended holiday season. C_t refers to the cumulative incidence estimated at each time step.

Model Calibration

The loglikelihood (LL) of observing the number of weekly ILI^+ reported in Shanghai during the 2017-2018 influenza season given the weekly number of ILI^+ estimated by the infection transmission model was defined as follows:

$$LL = \sum_{t=1}^{18} \log(NB(N_{ILI+}, rK_t, \sigma))$$

Where:

- N_{ILI+}refers to the weekly number of ILI^+;
- r is the reporting rate;
- K_t is the weekly number of ILI^+ estimated by the transmission model;
- σ is the over-dispersion of the negative binomial distribution;
- $NB(N_{ILI+}, rK_t, \sigma)$ is the negative binomial probability density function of observing N_{ILI+} from a negative binomial distribution of mean rK_t and over-dispersion σ.

We used MCMC with Metropolis-Hastings sampling to explore this likelihood and estimate the joint posterior distributions of the parameter vector (transmission risk, number of initial infections imported on December 4, the reporting rate, and the over-dispersion of the negative binomial distribution). We ran chains consisting of 1 million iterations (Fig. S10) and convergence was assessed by selecting multiple initial values of the parameters. This resulted in an average reporting rate of 0.45% (95% CI: 0.39-0.51%), average per-contact transmission rate of 0.0185 (95% CI: 0.0181-0.0189), 5,579.0 (95% CI: 3,979.9-7,557.0) infected individuals on average, and an average over-dispersion of 16.8 (95% CI: 7.4-30.2).

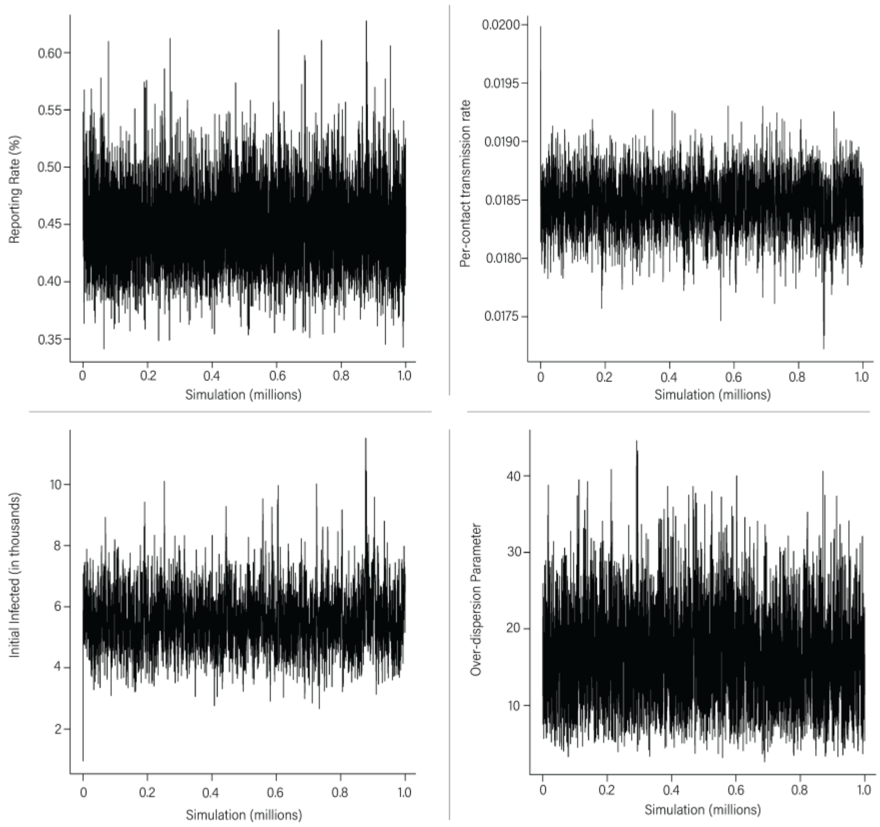

Figure S9. Trace plots of the estimated model parameters.
